# Impact of 5-Year Endoscopic Surveillance Intervals with Biopsy following Endoscopic Papillectomy for Ampullary Adenoma

**DOI:** 10.3390/jpm12010051

**Published:** 2022-01-05

**Authors:** Hoonsub So, Sung Woo Ko, Seung Hwan Shin, Eun Ha Kim, Do Hyun Park

**Affiliations:** 1 Department of Internal Medicine, Ulsan University Hospital, University of Ulsan College of Medicine, Ulsan 44030, Korea; hoon3112@gmail.com; 2 Department of Internal Medicine, The Catholic University of Korea, Eunpyeong St. Mary’s Hospital, Seoul 06591, Korea; gogo930@catholic.ac.kr; 3 Division of Gastroenterology, Department of Internal Medicine, University of Ulsan College of Medicine, Asan Medical Center, Seoul 05505, Korea; ssh881014@gmail.com (S.H.S.); sp98rn@gmail.com (E.H.K.)

**Keywords:** endoscopic snare papillectomy, adherence, recurrence, ampullary adenoma, pancreatitis

## Abstract

**Background:** Endoscopic snare papillectomy (ESP) has been established as a safe and effective treatment for ampullary adenomas. However, little is known about the optimal post-procedure follow-up period and the role of routine endoscopic surveillance biopsy following ESP. We aimed to evaluate patient adherence to a 5-year endoscopic surveillance and routine biopsy protocol after ESP of ampullary adenoma. **Methods:** We reviewed our prospectively collected database (*n* = 98), all members of which underwent ESP for ampullary lesions from January 2011 to December 2016, for the evaluation of long-term outcomes. The primary outcome was the rate of patient adherence to 5-year endoscopic surveillance following ESP. The secondary outcomes were the diagnostic yield of routine endoscopic biopsy, recurrence rate, and adverse events after endoscopic surveillance in the 5-year follow-up (3-month, 6-month, and every 1 year). **Results:** A total of 19 patients (19.4%) experienced recurrence during follow-up, all of these patients experienced recurrence within 3 years of the procedure (median 217 days, range 69–1083). The adherence rate for patients with sporadic ampullary adenoma were 100%, 93.5%, and 33.6% at 1, 3, and 5 years after ESP, respectively. The diagnostic yield of routine endoscopic biopsy without macroscopic abnormality was 0.54%. Pancreatitis occurred in four patients (4%, 3 mild, 1 moderate) after surveillance endoscopic biopsy without macroscopic abnormality. **Conclusions:** Given the low 5-year adherence rate and diagnostic yield of routine endoscopic biopsy with risk of pancreatitis, optimal surveillance intervals according to risk stratification (low grade vs. high grade adenoma/intramucosal adenocarcinoma) may be required to improve patient adherence, and routine biopsy without macroscopic abnormality may not be recommended.

## 1. Introduction

Endoscopic snare papillectomy (ESP) has been established as a safe and effective treatment for ampullary adenomas. To date, this treatment has largely replaced surgical ampullectomy [1]. Recent studies on ESP have focused on the efficacy, safety, and long-term outcomes of ampullary adenomas [2]. However, little is known about the optimal post-procedure follow-up period and the role of routine endoscopic surveillance biopsy for ampullary adenoma following ESP. The downside of ESP is the relatively high recurrence rate. The recurrence rate due to incomplete endoscopic resection by ESP is reported to be as high as 30% [3,4]. Large size, intraductal extension, piecemeal resection, and lack of awareness of residual lesions are factors for recurrence. Therefore, well-planned surveillance is necessary following ESP. The European Society of Gastrointestinal Endoscopy (ESGE) guideline recommends long-term surveillance with duodenoscopy within the first 3, 6, and 12 months, and then yearly for at least 5 years [5]. The American Society for Gastrointestinal Endoscopy (ASGE) does not suggest specific years of follow-up [6]; however, recent expert consensus advocates 5 years of surveillance [7]. The consensus also suggests that surveillance biopsy should be performed at macroscopic recurrence [7]. However, these suggestions are based on low-quality evidence. Five-year routine endoscopic surveillance may affect the burden on endoscopic resources without apparent benefits and unnecessary endoscopic routine biopsies performed in the endoscopic follow-up examination with the occurrence of pancreatitis. Therefore, we aimed to evaluate the impact of follow-up endoscopy and the role of regular endoscopic biopsy after ESP of ampullary adenoma through a retrospective study with a 5-year follow-up endoscopy protocol in our center.

## 2. Methods

### 2.1. Study Population

We reviewed our prospective collected database of patients who underwent ESP for ampullary lesions from January 2011 to December 2016 for the evaluation of long-term outcomes. The exclusion criteria were patients with (1) no available follow-up data and (2) no suitable indication for ESP. The data regarding the baseline characteristics (age, sex, underlying disease), clinical outcome (adverse event, follow-up endoscopy), and pathologic outcome (en bloc resection, diagnosis, size, resection margin) were collected and analyzed. The primary outcome was the rate of patient adherence to the 5-year follow-up endoscopy protocol. The secondary outcomes were the complete resection rate of ESP, recurrent rate of ampullary adenoma, and adverse events after surveillance biopsy in a 5-year follow-up (3-month, 6-month, and every 1 year). The Institutional Review Board of Asan Medical Center approved this study (2021-0112, approval date 25 January 2021). The study was conducted in accordance with ethical guidelines of the 1975 Declaration of Helsinki.

### 2.2. Endoscopic Technique

Computed tomography was performed in all patients prior to the procedure. An additional evaluation with endoscopic ultrasonography was performed in selected patients at the discretion of the endoscopist. A side-view endoscope (TJF-260V or JF-260V; Olympus Medical Co., Tokyo, Japan) was used for ESP. Normal saline was injected only at the anal side of the lesion followed by ESP. Stiff hexagonal snares (Captivator II, Boston Scientific, Malborough, MA, USA) with a diameter of 27 mm in most cases or a diameter of 13 mm in tumors less than 1 cm were used for ESP. A pre-cutting current (Endocut Q mode, effect 3) in the electrosurgical unit (ERBE, Tübingen, Germany) was used to transect the lesion. If en bloc resection was not possible, piecemeal resection and/or argon plasma coagulation (APC) ablation was performed. For cases with intraductal tumor extension, an additional snare papillectomy or APC ablation was performed. A pancreatic duct stent (Zimmon, single pig-tail stent, 5F in diameter, 3 cm in length, Cook Endoscopy, Bloomington, IN, USA) was inserted for the prevention of pancreatitis. Endoscopic sphincterotomy and/or biliary stent were performed at the discretion of an endoscopist. All procedures were performed by a single expert (D.H.P).

### 2.3. Definition

Complete resection was defined as en bloc resection with a clear resection margin. Piecemeal resection, indeterminate margins, and ductal involvement were also classified as incomplete resection. Recurrences were defined as newly pathologic diagnosed ampullary adenoma on a follow-up biopsy after ESP. Adverse events and their severity were classified according to the lexicon for endoscopic adverse events proposed by consensus guidelines [8]. Oozing controlled by endoscopic intervention without requiring transfusion during second-look endoscopy following ESP was graded as a mild adverse event. Pancreatitis was defined by at least a threefold increase in serum amylase and/or lipase coupled with abdominal pain.

### 2.4. Follow-Up Schedule

Follow-up endoscopies were scheduled at 3 months (for the removal of previously placed pancreatic stents during ESP), 6 months, 12 months, and subsequently at 1-year intervals from the procedure for a total of 5 years. A duodenoscope was used for routine endoscopic biopsy at the endoscopic resection site regardless of macroscopic recurrence (worrisome mucosal findings including nodularity). Without macroscopic lesions, two pieces of tissue were routinely obtained at the common bile duct (CBD) orifice side of the papillectomy scar, avoiding the pancreatic duct orifice. Patients requiring routine follow-up in familial adenomatous polyposis (FAP) following ESP were excluded for the evaluation of adherence to follow-up protocol. A detailed follow-up program was explained after immediate ESP and in each out-patient clinic to all the enrolled patients. Patients with follow-up loss were contacted by nurse-led telephone follow-up calls (E.H.K.) for any endoscopic follow-up with recurrence in other centers at the end of the 5-year follow-up.

### 2.5. Statistical Analysis

Descriptive statistics, including means, standard deviations, and percentages, were calculated. Categorical parameters are expressed as frequencies and proportions and compared using the chi-square test or Fisher’s exact test. We used the Kaplan–Meier method to estimate the cumulative adherence and recurrence. The data regarding relapsed patients were treated as censored, as a suitable follow-up schedule was applied for subsequent endoscopic therapy. The adherence rate was analyzed using the Kaplan–Meier method. An additional sub-group analysis with patients with complete resection was performed. All reported *p*-values are two-sided and a *p*-value of <0.05 was considered to indicate statistical significance. The data were analyzed using R program version 4.1.0 (R Foundation for Statistical Computing, Vienna, Austria, http://www.R-project.org, accessed on 2 October 2021).

## 3. Results

### 3.1. Baseline Characteristics

**Figure 1:**
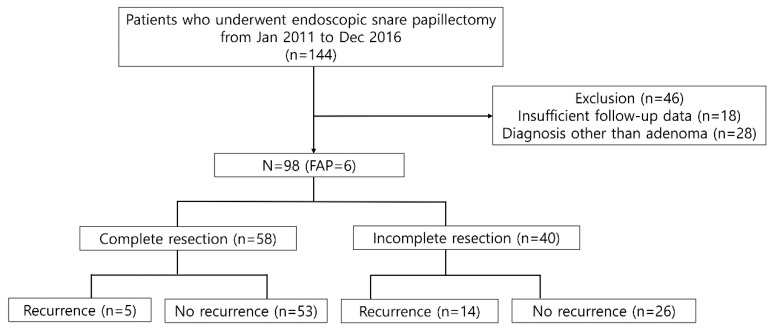
Flow chart of the patient inclusion process and recurrence rate according to resection margin after endoscopic snare papillectomy. FAP—familial adenomatous polyposis.

A total of 144 consecutive patients were screened during the study period. Notably, 46 patients were excluded due to the following reasons: (1) insufficient follow-up data (*n* = 18) and (2) no suitable indication for ESP (*n* = 28, pancreatic intraepithelial neoplasia 2 or chronic papillitis, heterotopic pancreas, neuroendocrine tumor, etc.) (Figure 1). Therefore, a total of 98 patients were included in the analysis. The median age was 56 years (interquartile range (IQR), 48–66 years), and 64 patients were men. Six patients were diagnosed with FAP. One had previous history of bile duct resection with hepaticojejunostomy. All patients did not show jaundice and they did not undergo routine endoscopic biliary sphincterotomy with biliary decompression (Table 1).

### 3.2. Pathologic Outcomes of ESP

The pathology size was median 12 mm (IQR, 8–18). The sizes were classified as smaller than 1 cm (*n* = 33), between 1–2 cm (*n* = 47), and over 2 cm (*n* = 18). The pathology was adenoma with low-grade dysplasia (*n* = 74), adenoma with high-grade dysplasia (*n* = 13), and intramucosal adenocarcinoma (*n* = 11). Complete resection was achieved in 58 (59.1%) patients. Incomplete resection was observed in 40 patients: positive resection margin (*n* = 9), difficult margin assessment (*n* = 31). The reasons for difficult margin assessment were as follows: indeterminate resection margin (*n* = 4), piecemeal resection (*n* = 7), CBD involvement (*n* = 15), main pancreatic duct (MPD) involvement (*n* = 2), thermal denaturation (*n* = 3). The results are summarized in Table 2.

### 3.3. Adverse Events of ESP

Bleeding or oozing during second-look endoscopy on the next day after ESP was the most common adverse event, occurring in 39 patients (39.8%). All these patients were treated successfully with endoscopic hemostasis, such as epinephrine injection or use of APC, without requiring angiographic or surgical intervention. The severity of bleeding in patients was mild except for 3 patients, who needed transfusions. Post-procedural pancreatitis and perforation following ESP occurred in 12 (12.2%, mild *n* = 10, moderate *n* = 2) patients and 2 (2.0%) patients, respectively. These patients were observed to recover without any sequelae, with supportive treatment only. The results are summarized in Table 2.

### 3.4. Long-Term Follow-Up Outcome and Adherence to the Surveillance Schedule

**Figure 2:**
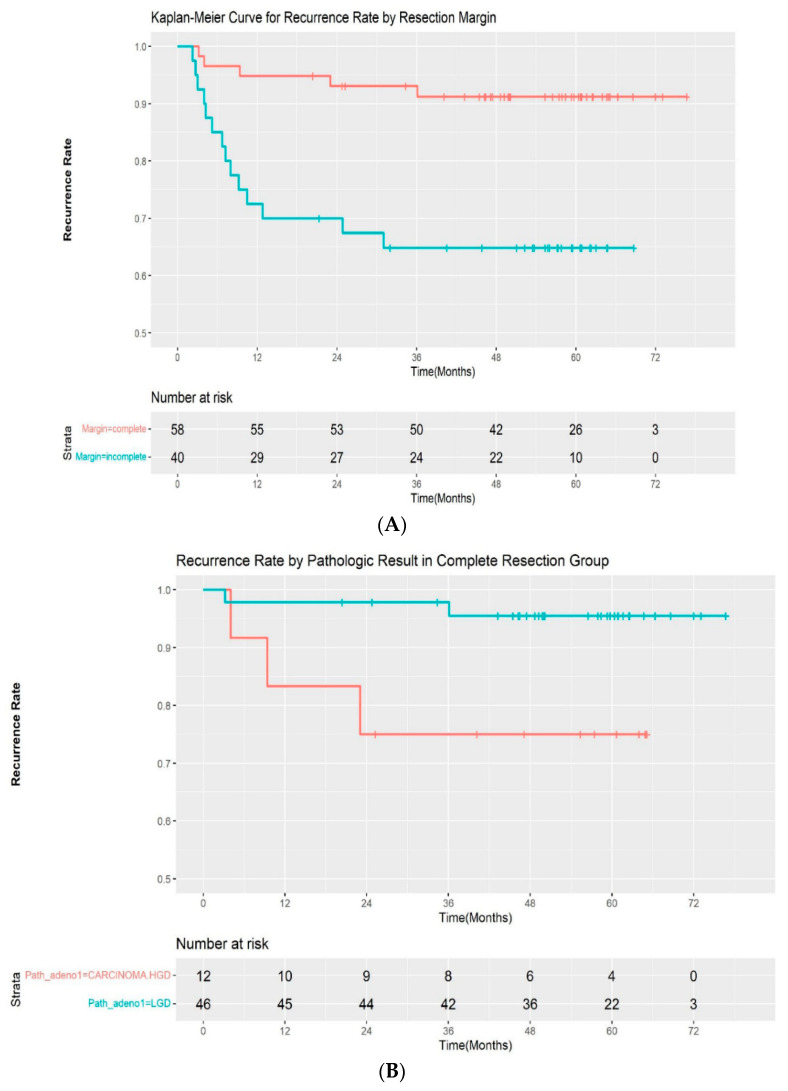

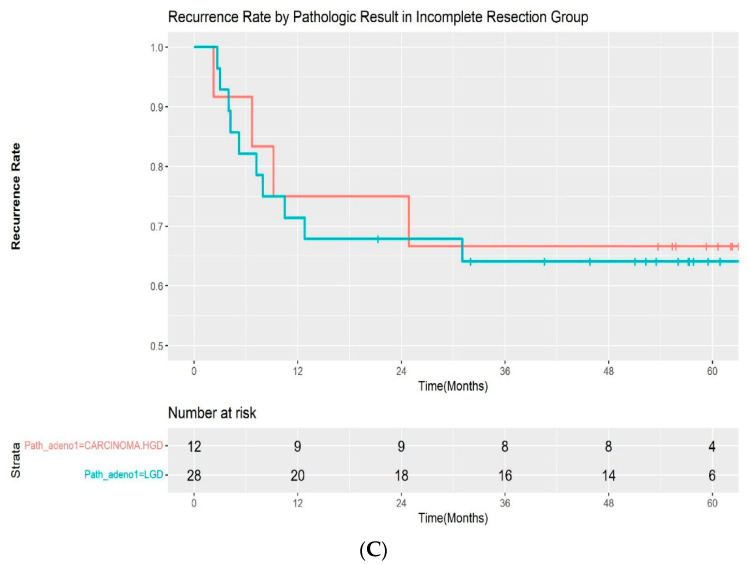
(**A**) Kaplan–Meier curve for the recurrence rate according to resection margin. (**B**) Kaplan–Meier curve for the recurrence rate according to pathologic result in the complete resection group. (**C**) Kaplan–Meier curve for the recurrence rate according to pathologic result in the incomplete resection group. LGD—low-grade dysplasia; HGD—high-grade dysplasia.

A total of 19 patients (19.4%) experienced recurrence during follow-up. All of the recurrences occurred within 3 years of the initial procedure (median: 217 days; range, 69–1083 days). Additionally, 5 of 58 (8.6%) patients with complete resection and 14 of 40 (35.0%) patients with incomplete resection experienced recurrences, respectively. The median time for recurrence was 282 days (range, 96–1083 days) in the complete resection group and 209 days (68–931 days) in the incomplete resection group. The recurrence rate was significantly different between the two groups by log-rank test (*p* < 00.001) (Figure 2A). A subgroup analysis was performed by pathologic outcomes. In the complete resection group, recurrences occurred in 2 of 46 (4.3%) patients with low-grade adenoma and 3 of 12 (25%) patients with high-grade dysplasia/intramucosal adenocarcinoma (Figure 2B). A log-rank test demonstrated that the difference was statistically significant (*p* = 0.02). However, it was not significantly different in the incomplete resection group, with a recurrence rate of 10 of 28 (35.7%) and 4 out of 12 (33.3%) in patients with low-grade dysplasia and high-grade dysplasia/intramucosal adenocarcinoma, respectively (*p* = 0.9) (Figure 2C). The recurrences were managed endoscopically with APC ablation in 11 cases, with subsequent ESP in 5 cases. One patient was referred for surgery, as adenoma with high-grade dysplasia was confirmed during follow-up. Recurrence was not treated in 2 patients due to the wishes of the patients. The median recurrence-free interval was 185 days (range, 81–1083) and 276 days (range, 68–746) for patients with low-grade adenoma and high-grade adenoma, respectively. No adenocarcinoma was observed in the recurred cases. The recurrence rate after initial ESP and subsequent treatment according to the resection margin are summarized in Table 3.

**Figure 3:**
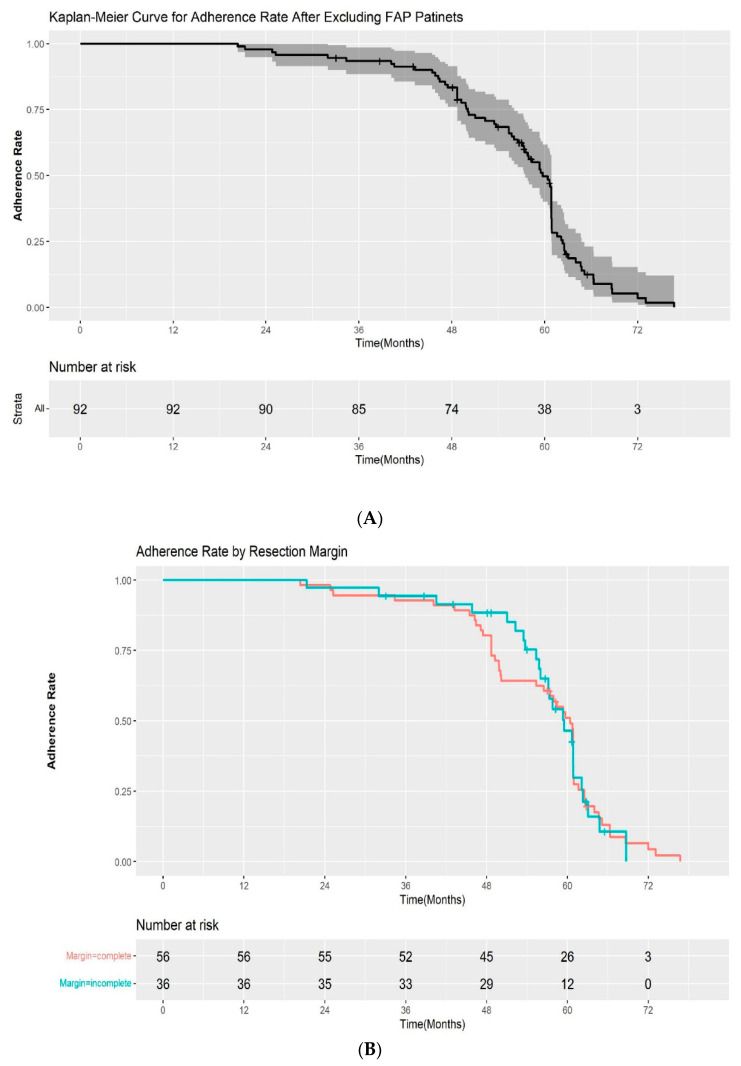

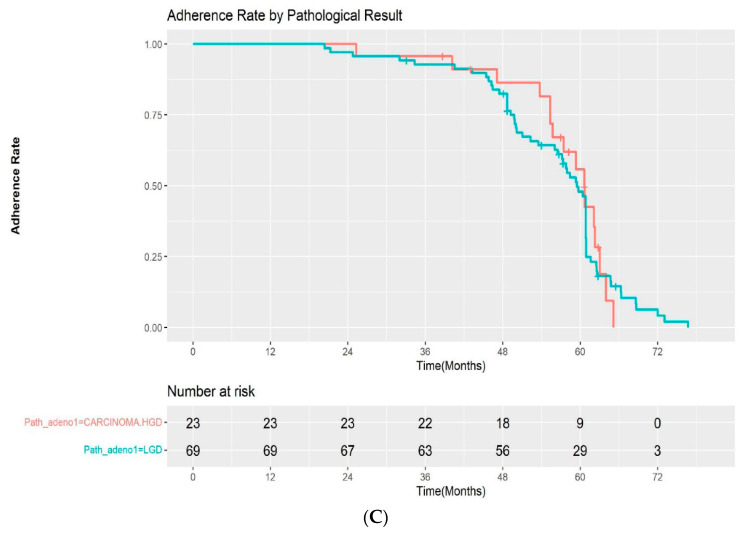
(**A**) Kaplan–Meier curve for the adherence rate to endoscopic surveillance after endoscopic snare papillectomy after excluding FAP patients. (**B**) Kaplan–Meier curve for the adherence rate to endoscopic surveillance after endoscopic snare papillectomy according to resection margin. (**C**) Kaplan–Meier curve for the adherence rate to endoscopic surveillance after endoscopic snare papillectomy according to pathological results. HGD—high-grade dysplasia; LGD—low-grade dysplasia.

The adherence rate for all study cohorts except the patients with FAP were 100%, 93.5%, and 33.6% at 1, 3, and 5 years after ESP, respectively, in the Kaplan–Meier analysis (Figure 3A). Among the patients with a complete resection margin, the adherence to scheduled follow-up was as follows: 100% at 1 year, 92.9% at 3 years, and 33.4% at 5 years. In the incomplete resection group, the rates of adherence to scheduled follow-up were 100%, 94.4%, and 34.1% at 1, 3, and 5 years, respectively. The difference in the adherence rate between the two groups was not statistically significant by log-rank test (*p* = 0.2) (Figure 3B). We also evaluated the adherence rate according to the pathological results after ESP (high-grade dysplasia/adenocarcinoma vs low-grade dysplasia). The 1, 3, and 5-year adherence rates after ESP were 100%, 95.7%, 42.5%, and 100%, 92.7%, and 31.4% for patients with high-grade dysplasia/intramucosal adenocarcinoma and low-grade dysplasia, respectively. Although the adherence rate at 5 years was higher in the high-grade dysplasia/adenocarcinoma group, the difference was also not statistically significant (*p* = 0.9) (Figure 3C).

### 3.5. Impact of Routine Surveillance without Macroscopic Abnormality

Two recurrences were diagnosed after biopsy without macroscopic abnormalities. The yield of routine surveillance endoscopic biopsy without macroscopic abnormality was 0.54% (2/365, recurred case by surveillance biopsy without macroscopic abnormality/the number of endoscopies with surveillance biopsy without macroscopic abnormality). Those two recurrences were diagnosed as high-grade dysplasia by ESP: one with complete resection, one with an indeterminate resection margin. The timings of the recurrences were 2 years and at 6 months after the procedure, respectively.

Pancreatitis occurred in 4 patients (4%, *n* = 4/98 as per-protocol analysis) after surveillance endoscopic biopsy without macroscopic abnormality. All biopsy results were duodenitis and the timings of pancreatitis were at 2 years (*n* = 2), 3 years (*n* = 1), 5 years (*n* = 1) after EP. They were initially diagnosed with adenoma with low-grade dysplasia (*n* = 2), high-grade dysplasia (*n* = 1), and intramucosal adenocarcinoma (*n* = 1) by ESP; three with complete resection, one with indeterminate resection margin. The grade of severity was mild (*n* = 3) and moderate (*n* = 1). These patients recovered by conservative treatment.

## 4. Discussion

ESP has shown its safety and efficacy for treating ampullary adenoma; however, there is still a lack of evidence regarding its surveillance protocols or additional treatment. Our study’s adherence rate was as high as 93.5% up to 3 years; it declined to 33.6% thereafter, however, as a significant number of patients were lost to follow-up. Therefore, making all patients with ampullary adenoma and ESP undergo follow-up endoscopy for 5 years might be a formidable task in the real world, especially for those with neoplasms, which carries a low risk of malignancy, such as low-grade adenoma. Furthermore, regular surveillance up to 5 years without apparent clinical benefit could be a burden to endoscopic resources in the open-access endoscopic suite. 

Several studies on the early recurrence of ampullary adenomas after ESP have been reported. S. Li et al. reported the outcomes of ESP in 110 patients with ampullary neoplasms; 13 patients experienced recurrence. Among them, 92.3% (12/13) of patients relapsed within 3 years [9]. Van der Wiel et al. [10] and K. Takahashi et al. [11] also evaluated the time to recurrence after ESP; 90% (9/10) and all patients (13/13) demonstrated recurrences within 3 years, respectively. On the contrary, there are also recent papers reporting late recurrences [12,13,14], i.e., 5 years after ESP. A. Tringali et al. reported one late recurrence as adenocarcinoma 73 months after ESP in which a diagnosis of high-grade dysplasia with piecemeal resection was made [15]. However, the clinical and pathological characteristics of patients with late recurrences have not been evaluated in those studies. Recent studies on the time to recurrence after ESP including our study are summarized in Table 4. Considering that most recurrences appeared within 3 years after ESP in previous studies and this one, the clinical impact of 5-year routine surveillance for all patients following ESP may be uncertain. In this study, patients with advanced pathologies experienced a much higher recurrence rate than those with low-grade dysplasia, even after complete resection. However, after incomplete resection, the grade of dysplasia did not affect the recurrence. Therefore, strategies should be tailored for individuals based on pathologic results. For example, for patients with complete resection with low-grade dysplasia, follow-up endoscopic surveillance can be stopped at 3 years following ESP, subsequent to which on-demand endoscopy is sufficient. Additionally, in patients with incomplete resection and/or advanced pathology, further surveillance beyond 3 years might be warranted. This could reduce the burden on endoscopic resources, patients’ risk for possible biopsy-induced pancreatitis, and medical costs.

In terms of surveillance biopsy, four cases of pancreatitis occurred. Even though we tried to obtain a biopsy at the CBD orifice side, away from the MPD orifice, pancreatitis was not completely avoidable. Two microscopic recurrences by routine surveillance biopsy without macroscopic abnormality occurred. The yield of routine surveillance endoscopic biopsy without macroscopic abnormality was 0.54%. Considering a low yield of routine surveillance biopsy without macroscopic abnormality and the risk of pancreatitis, it might be reasonable to only biopsy when there is a macroscopic abnormality. Recent expert consensus also recommends taking a biopsy when there is a macroscopic abnormality [7].

The timing of pancreatitis after biopsy occurred after 2 years of ESP in this study. Several factors associated with acute pancreatitis after ESP have been reported, such as jaundice, large tumors, and dilated MPD. However, pancreatitis after biopsy at the ESP scar is a different situation and factors related to pancreatitis are not reported.

The strength of this study is that we educated all the enrolled patients regarding the importance of the follow-up schedule before starting surveillance, and the end of 5-year follow-up was achieved in all enrolled patients using protocol basis or telephone follow-up call for the evaluation of the impact of 5-year routine endoscopic surveillance with biopsy. Therefore, the result of the adherence reflects the real-world clinical situation.

There are also several limitations. There is an inherent limitation in the study’s retrospective design, including the selection bias. Approximately 30% of patients were observed with difficult margin assessment. Different results in the adherence rate for follow-up protocol after ESP could have been obtained depending on pathological result, race, social-economic status, distance from home, and different medical reimbursement systems, among others. Furthermore, the number of included patients was relatively small to warrant revising the current 5-year follow-up recommendation.

## 5. Conclusions

In conclusion, given low 5-year adherence rate and diagnostic yield of routine endoscopic biopsy with risk of pancreatitis after ESP for ampullary adenomas, optimal surveillance intervals according to risk stratification may be required to improve patient surveillance adherence rate, and routine biopsy without macroscopic abnormality may not be recommended. Personalized surveillance strategies based on pathologic outcomes of ESP (complete resection with low-grade dysplasia vs. incomplete resection and/or high-grade adenoma/intramucosal adenocarcinoma) could reduce the burden on endoscopic resources, patient’s risk for possible biopsy-induced pancreatitis, and medical costs.

## Figures and Tables

**Table 1:** Baseline characteristics of the included patients.

	*n* = 98
Age, median (IQR) (y)	56 (48–66)
Male sex, *n* (%)	64 (65.3)
Familial adenomatous polyposis, *n* (%)	6 (6.1)
Presenting symptoms, *n* (%)	
Incidental finding on endoscopy	73 (74.5)
Incidental finding on CT	4 (4.1)
FAP surveillance	6 (6.1)
Overt symptom (e.g., abdominal pain)	15 (15.3)
Size, median (IQR) mm	12 (8–18)
<1 cm, *n* (%)	33 (33.7)
≥1 cm and <2 cm, *n* (%)	47 (48.0)
≥ 2 cm, *n* (%)	18 (18.3)

IQR: Interquartile range; CT: Computed tomography; FAP: Familial adenomatous polyposis.

**Table 2:** Pathological outcomes and adverse events after endoscopic papillectomy.

	*n* = 98
Diagnosis, *n* (%)	
Adenoma with low-grade dysplasia	74 (75.5)
Adenoma with high-grade dysplasia	13 (13.3)
Adenocarcinoma	11 (11.2)
Complete resection, *n* (%)	
Clear resection margin with en bloc	58 (59.1)
Incomplete resection, *n* (%)	
Resection margin involvement	9 (9.2)
Deep margin positive	7 *
Lateral margin positive	1
Both deep and lateral margin positive	1
Difficult margin assessment, *n* (%)	31 (31.7)
Indeterminate clear resection margin	4
Piecemeal resection	7
CBD involvement	15
MPD involvement	2
Thermal denaturation	3
Adverse events, *n* (%)	
Bleeding	39 (39.8)
Mild	36
Moderate	3
Pancreatitis	12 (12.2)
Mild	10
Moderate	2
Perforation	2 (2.0)

* In one patient, resection was in a piecemeal manner, resulting in a positive deep resection margin. CBD—common bile duct, MPD—main pancreatic duct.

**Table 3:** Recurrence rate after initial endoscopic papillectomy and subsequent treatment according to resection margin.

	Complete Resection (*n* = 58)	Incomplete Resection (*n* = 40)
Recurrence, *n* (%)	5 (8.6%)	14 (35%)
Median recurrence free period, days (range)	282 (96–1083)	209 (68–931)
Treatment for recurrence		
Subsequent ESP	2	3
APC ablation	3	8
Surgery	0	1 *
Observation	0	2

APC—argon plasma coagulation; ESP—endoscopic snare papillectomy. * Underwent subtotal stomach preserving pancreaticoduodenectomy due to recurrence of high-grade dysplasia.

**Table 4:** Summary of recurrence rate and time to recurrence within 3 years after endoscopic snare papillectomy in recent studies, including our study.

Author, Year	Patient, *n*	Complete Resection	Adenoma with HGD/Carcinoma	Follow Up Period, Month	Recurrence Rate	Time to Recurrence, Month	Recurrence Rate within 3 Years *
S.Li, 2019 [9]	110	80.0% (88/110)	21.8% (24/110)/ 20.9% (23/110)	NA	11.8% (13/1)10	16.28 (6–132)	92.3% (12/13)
van der Wiel, 2019 [10]	87	47.1% (41/87)	0.0% (0/87)/31.0% (27/97)	18.6 (7.6–39.5) †	11.5% (10/87)	13.1 (4.6–33.1) mo †	90% (9/10)
A.Sakai, 2019 [16]	45	46.7% (21/45)	NA/26.6% (12/45)	27.1 (3.0–133.4)	8.9% (4/45)	3.1 (1.0–6.3) mo	100% (4/4)
N.Sahar, 2020 [14]	161	83% (106/128)	NA/1.2% (2/161)	30 (6–283)	7% (12/161)	36 (12–138)	NA
J.A.Fritzsche, 2020 [12]	259	59.1% (153/259)	15.4% (45/259)/14.3% (37/259)	40 (25.7–68) †	15.6% (24/154) ‡	29 (14.7–59) †	NA
S.Muro, 2021 [13]	46	41.3% (19/46)	NA/4.3% (2/46)	63 (1–150)	15.2% (7/46)	80 (7–123)	NA
R.Lee, 2021 [17]	53	56.6% (30/53)	43.4% (23/53)/3.2% (7/53)	30 (6–104)	32.7% (16/63)	9 patients: 3.9 3 patients: 7 4 patients: 25.3	NA
K.Takahashi, 2021 [11]	96	82.3% (79/96)	NA/35.4% (34/96)	55 (6–216)	13.5% (13/96)	3 (1–36)	100% (13/13)
This study, 2021	98	59.1% (58/98)	13.3% (13/98)/11.1% (11/98)	58.1 (49.3–61.5) †	19.3% (19/98)	7.2 (4.0–11.6) †	100% (19/19)

* Proportion of patients who relapsed within 3 years among all relapsed patients. Follow-up period and time to recurrence are expressed as median (range), except † median (interquartile). ‡ Recurrence rate was evaluated only for patients with at least 1-year follow-up. HGD—high-grade dysplasia.

## Data Availability

The data presented in this study are available on request from the corresponding author.
